# Transcriptomic Responses of the Endangered Endemic Fish *Aspiorhynchus laticeps* to Salinity–Alkalinity and Water Flow Stress

**DOI:** 10.3390/ani16152281

**Published:** 2026-07-23

**Authors:** Huanhuan Wang, Liting Yang, Changcai Liu, Wenxia Cai, Yong Song, Xuyuan Lin, Peng Chen, Zhen Sun, Sadia Bibi, Xiao Liang, Shengao Chen

**Affiliations:** 1 College of Life Science and Technology, State Key Laboratory Incubation Base for Conservation and Utilization of Bio-Resource in Tarim Basin, Tarim Research Center of Rare Fishes, Tarim University, Alar 843300, China; 10757242162@stumail.taru.edu.cn (H.W.); 10757231127@stumail.taru.edu.cn (L.Y.); 107572025416@stumail.taru.edu.cn (C.L.); 10757252199@stumail.taru.edu.cn (W.C.); songyongdky@126.com (Y.S.); i107572025413@stumail.taru.edu.cn (S.B.); 2 Xinjiang Production and Construction Corps Aquatic Technology Promotion Station, Urumqi 830000, China; btsc229@sina.com; 3 Xinjiang Uygur Autonomous Region Aquatic Development Center (Xinjiang Uygur Autonomous Region Aquatic Scientific Research Institute), Urumqi 830000, China; cpeng11@sina.com; 4 East China Sea Fisheries Research Institute, Chinese Academy of Fishery Sciences, Shanghai 200090, China; sunzhen@ecsf.ac.cn; 5 Zhejiang Key Laboratory of Coastal Biological Germplasm Resources Conservation and Utilization, State Key Laboratory for Quality and Safety of Agro-Products, Institute of Hydrobiology, Zhejiang Academy of Agricultural Sciences, Hangzhou 310021, China

**Keywords:** *Aspiorhynchus laticeps*, salinity-alkali stress, water flow, liver transcriptome, adaptive immunity, energy homeostasis

## Abstract

**Simple Summary:**

To elucidate the poorly understood adaptive mechanisms of *Aspiorhynchus laticeps,* a critically endangered fish endemic to Xinjiang’s Tarim Basin, under extreme aquatic stress, this study aimed to unravel the evolutionary adaptation patterns of plateau freshwater fish to environmental pressures. By integrating ecological experiments with transcriptome sequencing, we exposed *A. laticeps* to varied salinity–alkalinity and flow regimes for comparative analysis. We identified 1847 differentially expressed genes that were significantly enriched in TNF/NF-κB immune signaling and metabolic pathways. Experimental evidence confirmed that high saline–alkali stress activates these pathways and modulates target genes to enhance stress tolerance, whereas flow fluctuations regulate energy metabolism through distinct functional genes. This research provides molecular insights for artificial habitat regulation and population conservation of *A. laticeps* in the Tarim River, and offers a scientific foundation for endangered fish protection, germplasm improvement, and ecological rehabilitation in arid alpine zones.

**Abstract:**

To understand the adaptive evolution of endangered plateau freshwater fishes to environmental stress and to better explore the underlying mechanisms in *Aspiorhynchus laticeps*—a critically endangered fish endemic to the Tarim Basin, Xinjiang, China—a combination of ecological experiments and transcriptome sequencing (RNA-seq) technology was used to study the differences in gene expression patterns among individuals under different salinities and flow conditions. This experiment included four treatment groups (CON, H-SA-S, L-SA, L-SA-S). *A. laticeps* specimens with an average weight of 2.92 ± 0.62 g and a body length of 58.22 ± 5.10 mm were selected, with three biological replicates for a 96 h combined stress treatment. Moreover, the relationships between these differences and the aquatic environment were analyzed. A total of 1847 differentially expressed genes (DEGs), including 935 upregulated genes and 912 downregulated genes, were identified under different aquatic environment stress modes. GO and KEGG enrichment analyses revealed that TNF signal transduction, the NF-κB pathway, and metabolic regulation were significantly enriched among the DEGs (*p* < 0.05). High salinity–alkali stress significantly activates the TNF/NF-κB pathway, regulates *MST1*, *LOC107702867*, *LOC113110979* and other genes to enhance the body’s resistance; water flow changes mainly regulate energy metabolism through genes such as *NEHOM01_1600* and *gptl*. These findings provide an important scientific basis for the ecological adaptability, protection, and proliferation of endemic and endangered fish in China, as well as for germplasm innovation to address ecological deterioration in plateau fishes in alpine and arid areas. This study provides a molecular-level theoretical foundation for artificial habitat regulation and the conservation of endangered *Aspiorhynchus laticeps* populations in the Tarim River.

## 1. Introduction

As an important and strategic basic industry worldwide, fisheries not only ensure the supply of high-quality protein for human consumption but also play a key role in maintaining the ecological balance of aquatic environments and promoting regional economic development [1]. However, under the dual stresses of global climate change and intensifying human activities, aquatic ecosystems are undergoing unprecedented changes [2]. According to the latest report of the United Nations Food and Agriculture Organization [3], the salinized area of inland waters worldwide has expanded by 23% compared with that at the end of the twentieth century, and nearly 40% of freshwater fish populations have shown a significant decline [4,5]. This ecological crisis is particularly severe in the waters of northwest China, which directly threatens the distribution range and survival of plateau-specific fishes, represented by *Aspiorhynchus laticeps*.

*A. laticeps*, which belongs to Cyprinidae, Schizothoracinae, and *Aspiorhynchus*, is the sole species within its genus [6,7]. It is a large predatory fish, as well as the largest schizothoracinae fish in China and the flagship species of the Tarim River system. Moreover, *A. laticeps* has high economic value and scientific research value [8]. Once widely distributed across the entire Tarim River basin, the wild population of this top predatory cyprinid has declined by 99% over the past three decades, and extant populations are now restricted to the Kizil Reservoir–Bianxie Fish Provincial Nature Reserve in Baicheng County, Xinjiang, with an official Endangered (EN) conservation status [9]. As the only species in a monotypic genus and the largest schizothoracin fish endemic to northwest China, *A. laticeps* possesses a specialized osmoregulatory system and an obligate carnivorous feeding habit, making it an ideal model organism for plateau fish environmental adaptation research [10,11].

Previous stress physiology studies on *A. laticeps* have mainly focused on single salinity–alkalinity stress challenges [12]. No liver transcriptome profiling study has yet characterized the molecular regulatory networks in response to combined salinity–alkali and hydrodynamic water flow stress, and the core adaptive pathways and key functional genes remain uncharacterized [13]. This study fills this research gap and provides original molecular evidence for the conservation of plateau schizothoracin fish. Multiple transcriptome studies on Tarim River schizothoracin fishes published in recent years have reported that salinity–alkali stress severely disturbs hepatic immune and metabolic homeostasis; however, few studies have integrated hydrological flow factors into multi-stress omics analysis [14,15].

To clarify the molecular mechanism by which *A. laticeps* responds to environmental stress, this study used the Illumina NovaSeq 6000 platform and systematically established the gene regulatory network underlying the combined salinity–alkalinity and flow velocity stress response. We compared gene expression profiles under different saline–alkali stress and flow conditions, and identified key genes involved in environmental adaptation. This study is innovative in its combination of genomics and conservation biology to reveal, for the first time, the molecular adaptation strategy of this plateau fish under the stresses of complex aquatic environments. The results provide a scientific basis for developing precise conservation strategies for endangered species and guiding ecological restoration in river basins. Furthermore, this study offers new insights for aquatic biodiversity conservation under global climate change.

## 2. Materials and Methods

### 2.1. Sample Collection

**Figure 1:**
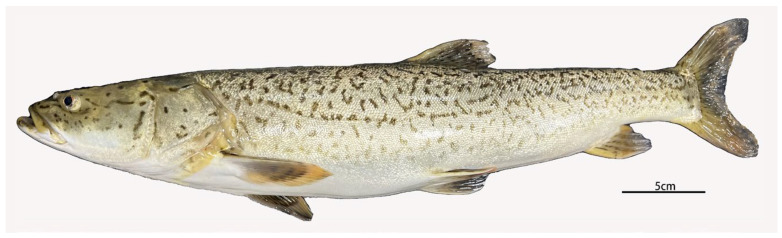
Morphological characteristics of *Aspiorhynchus laticeps*.

The experimental fish were selected from the original *A. laticeps* seed farm in Aksu, Xinjiang (Figure 1). *A. laticeps* specimens selected had an average weight of 2.92 ± 0.62 g, an average body length of 58.22 ± 5.10 mm, and there were 7 fish per tank. The fish were transported to the laboratory in oxygenated bags and underwent a 14-day acclimation period in a laboratory aquarium (60 × 30 × 45 cm).

The aquaculture water was continuously aerated for over 48 h. The water temperature was 21 ± 1 °C, salinity was 0.54 ppt, and the alkalinity was 2 mmol L^−1^, meeting the fishery water quality standard. The drugs used in the experiment were all analytically pure (AR). The salinity was adjusted using sodium chloride (NaCl, Nanjing Chemical Reagent Co., Ltd., Nanjing, China), and the alkalinity was adjusted using sodium bicarbonate (NaHCO_3_, Nanjing Chemical Reagent Co., Ltd., Nanjing, China).

### 2.2. Experimental Design

The 96 h acute toxicity test was performed according to Erhirhie et al. [16]. Based on the 96 h LC_50_ and safe concentration, three experimental groups and one control group were established.

CON group: Freshwater static water (salinity = 0.54 ppt, alkalinity = 2 mmol L^−1^, no water flow);

L-SA group: Low salinity–alkali static water (salinity = 3 ppt, alkalinity = 8 mmol L^−1^, no water flow);

L-SA-S group: Low salinity–alkali + high water flow (salinity = 3 ppt, alkalinity = 8 mmol L^−1^, continuous water flow);

H-SA-S group: High salinity–alkali + high water flow (salinity = 10 ppt, alkalinity = 18 mmol L^−1^, continuous water flow).

The solution concentration required for the experiment was configured in advance, with 10 L in each experimental tank. After standing for 24 h, the salinity was measured and corrected with a salinometer (Hanna 931101, Hanna Instruments, Woonsocket, RI, USA), and the alkalinity was measured and corrected with an acidity meter (Leima pH-3C, INESA Scientific Instrument Co., Ltd., Shanghai, China). Feeding was stopped 24 h before the start of the experiment. The water temperature was maintained at 21 °C, the mixture was continuously aerated (Sensen pump 0.75~1.1 kW), and the water was changed once a day. The amount of water exchanged was 1/3 of the total volume of the water body, and the water quality was kept clear and clean.

### 2.3. Sampling

The fish were divided into 4 groups: the control group (CON), the high-salinity–alkali + high water flow treatment group (H-SA-S), the low-salinity–alkali treatment group (L-SA), and the low-salinity–alkali + high water flow treatment group (L-SA-S). All group abbreviations are defined here upon first text appearance.

Each group contained 20 fish with three biological replicates per group (6–7 fish per replicate tank).

After 96 h of stress exposure, three fish were randomly selected from each group to be dissected quickly under sterile conditions with sterile knives and scissors, and liver samples were quickly extracted. The tissue samples were washed with normal saline and weighed, transferred to an enzyme-free EP tube, and frozen in liquid nitrogen. Some of the samples were stored at −80 °C for subsequent transcriptome analysis.

Three samples were collected from each group for high-throughput transcriptome sequencing. Before sampling, the fish were anesthetized with MS-222 and dissected. All experiments in this study were approved by the Ethics Committee of Tarim University. All animal experiments performed in this study were approved by the Science and Technology Ethics Committee of Tarim University (Approval No. 2023027, approval date: 25 June 2023).

### 2.4. RNA Extraction, Library Construction and Sequencing

Total RNA integrity and purity were assessed using a NanoDrop 2000 (Thermo Fisher Scientific, Wilmington, DE, USA) and an Agilent 2100 Bioanalyzer (Agilent Technologies, Inc., Santa Clara, CA, USA). Only RNA samples with OD260/OD280 ratios of 1.8–2.1 and RIN > 7.0 were retained for library construction. mRNA enrichment, fragmentation, cDNA synthesis, end repair, A-tailing, adapter ligation, and library PCR amplification were performed according to standard Illumina NovaSeq 6000 protocols without customized modifications. Qualified libraries were sequenced on the Illumina NovaSeq 6000 platform (Illumina, Inc., San Diego, CA, USA).

### 2.5. RNA Sequencing Data Analysis

After removing reads containing adapters, poly-N sequence, and low-quality sequence from the original data, the clean reads were sequenced. The efficiency of sequence alignment is affected not only by the quality of sequencing data but also by the assembly methods used for the specific reference genome, the taxonomy of the reference genomes, and the sample sequencing. This efficiency can be measured to evaluate whether the selected reference genome meets the needs of data analysis. Moreover, the Q20, Q30, and GC contents, and repetitive sequence content of the clean data were calculated. All downstream analyses used only clean, high-quality data. The clean reads were subsequently mapped to the reference genome sequence. Only fully matched or mismatched reads were further analyzed and annotated according to the reference genome.

### 2.6. Identification and Screening of Differentially Expressed Genes

After the new transcripts were assembled, the TPM (transcripts per million) method was used to calculate the gene expression level, and DESeq2 v1.36.0 software was used to analyze the differences in gene expression. The false discovery rate (FDR) was obtained by adjusting *p*-values. Differentially expressed genes (DEGs) were identified using the thresholds of |log_2_FC| ≥ 1 and FDR < 0.05.

For convenience of comparison, the difference multiple was presented as log_2_FC. Larger absolute values of log_2_FC indicate more distinct differences in the expression of genes with smaller FDR values between the two groups of samples.

The screened DEGs were annotated according to multiple public databases, including Cluster of Orthologous Groups of proteins (COG), Gene Ontology (GO), Kyoto Encyclopedia of Genes and Genomes (KEGG), EuKaryotic Orthologous Groups (KOG), NCBI Non-redundant Protein Database (NR), Protein Family Database (Pfam), Manually Annotated Protein Sequence Database (Swiss-Prot), and Automated Annotated Protein Database (TrEMBL). The distribution of DEGs in different databases was analyzed, and the numbers of DEGs with different functions and their biological functions are displayed in the form of graphics and tables. 

### 2.7. Real-Time Quantitative PCR (RT-qPCR) Validation

To validate the transcriptome data, six DEGs were selected for RT-qPCR. cDNA was synthesized from the same RNA samples used for sequencing. Each sample was analyzed in triplicate, with EF-1α as the internal reference gene. Primers were designed using NCBI Primer-BLAST (https://blast.ncbi.nlm.nih.gov/) (Table 1). The relative expression formula was standardized as R = 2^−ΔΔCt^.

### 2.8. Statistical Analysis

Statistical analysis was performed using SPSS 20.0 software (IBM Corp., Armonk, NY, USA). The experimental data are expressed as the means ± standard deviations (SD). The statistical significance of the differences between groups was tested by one-way ANOVA, and correction for multiple comparisons was performed using the Tukey post hoc method. Three independent replicates were performed for all experiments, and *p* < 0.05 was used as the criterion to indicate statistical significance.

## 3. Results

### 3.1. Transcriptome Sequencing and Assembly

Table 2 summarizes the transcriptome sequencing indicators of 12 mRNA libraries (CON1, CON2, CON3, H-SA-S1, H-SA-S2, H-SA-S3, L-SA1, L-SA2, L-SA3, L-SA-S1, L-SA-S2, L-SA-S3) from the liver of *A. laticeps*. The 12 transcriptome libraries obtained 38,823,066, 39,523,252, 54,618,086, 55,286,312, 58,637,532, 37,406,962, 36,077,826, 41,263,062, 56,036,160, 38,426,334, 38,304,982, 36,847,090, respectively. The Q30 base mass values of the four sequencing libraries of CON, H-SA-A, L-SA, and L-SA-S ranged from 86% to 90%, Q20 ranged from 95% to 90%, and GC content ranged from 43% to 48%. The above quality control indicators showed that all sequencing libraries met the strict criteria for reliable transcriptome analysis (Table 2).

The results of Unigene database annotation were as follows: 37,202 (78%) genes were annotated in the NR database, 28,920 (60%) genes were annotated in the SwissProt database, 17,337 (36%) genes were annotated in the KEGG database, 21,283 (44%) genes were annotated in the KOG database, 6422 (13%) genes were annotated in the COG database, 18,241(38%) genes were annotated in the GO database, and 28,806 (60%) genes were annotated in the Pfam database (Table 3).

### 3.2. Differential Expression Analysis

**Figure 2:**
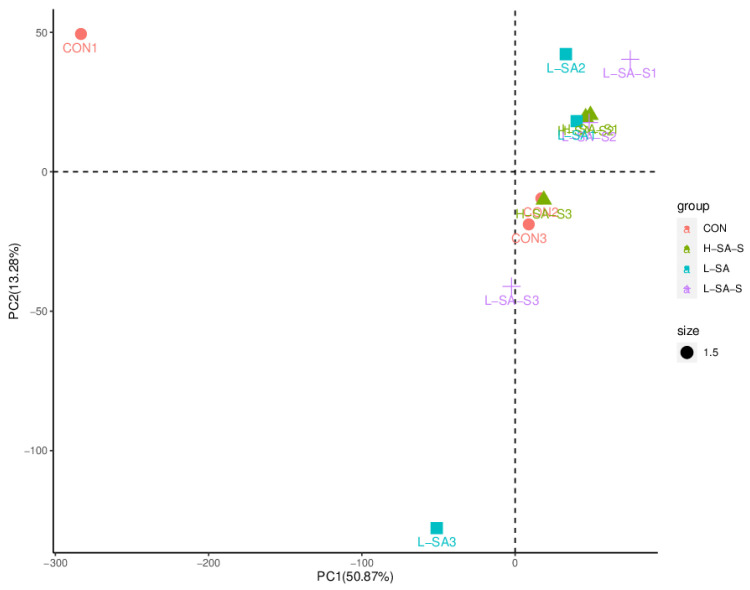
PCA of the transcriptome sequencing results of the different salinity and flow rate treatment groups. PC1 (50.87% variance) reflects transcriptional variation induced by salinity–alkali concentration gradients; PC2 represents gene expression fluctuation driven by water flow velocity differences. Samples within identical groups are clustered tightly, verifying reliable repeatability of biological replicates. Pronounced separation means a remarkable transcriptional difference between L-SA-S and control.

**Figure 3:**
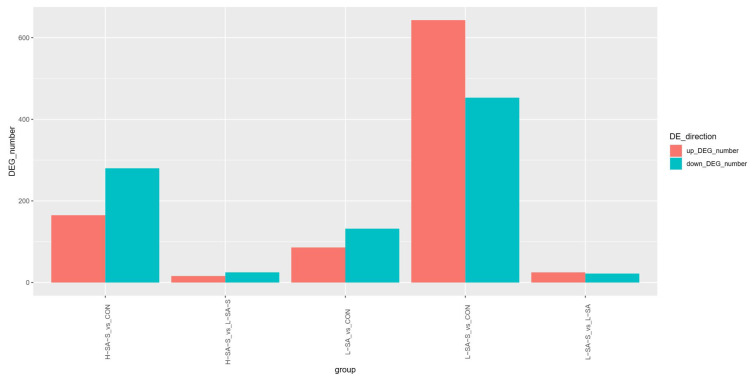
Bar chart statistics of upregulated and downregulated DEGs from five pairwise group comparisons.

Transcriptome analysis revealed significant differences in gene expression between the experimental groups. Principal component analysis (PCA) revealed that the samples presented a close intragroup clustering distribution, indicating that the biological replicates had good reproducibility (Figure 2). The combined low salinity–alkali and high flow treatment group (L-SA-S) showed the most pronounced separation from the control group, while the single low salinity–alkali group (L-SA) exhibited minimal transcriptional changes. Comparative analysis revealed a total of 1847 differentially expressed genes (DEGs, screening criteria: |log_2_FC| ≥ 1, FDR < 0.05) across different salinity and water flow conditions, including 935 upregulated genes and 912 downregulated genes (Figure 3).

In this study, clean reads were mapped to a custom-built cDNA library of 10 reference transcripts using a systematic comparison strategy. Five pairwise comparisons were made: H-SA-S vs. control; H-SA-S vs. L-SA-S; L-SA vs. control; L-SA-S vs. control; and L-SA-S vs. L-SA. There were 544 DEGs (165 upregulated and 280 downregulated) in the H-SA-S vs. control; 41 DEGs (16 upregulated and 25 downregulated) in the H-SA-S vs. L-SA-S; 1096 DEGs (643 upregulated and 453 downregulated) in the L-SA-S vs. control; 47 DEGs (25 upregulated and 22 downregulated) in the L-SA vs. control, and 218 DEGs (86 upregulated and 132 downregulated) in the L-SA-S vs. L-SA (Figure 3). L-SA-S vs CON had the maximum number of DEGs, representing the strongest transcriptional divergence.

**Figure 4:**
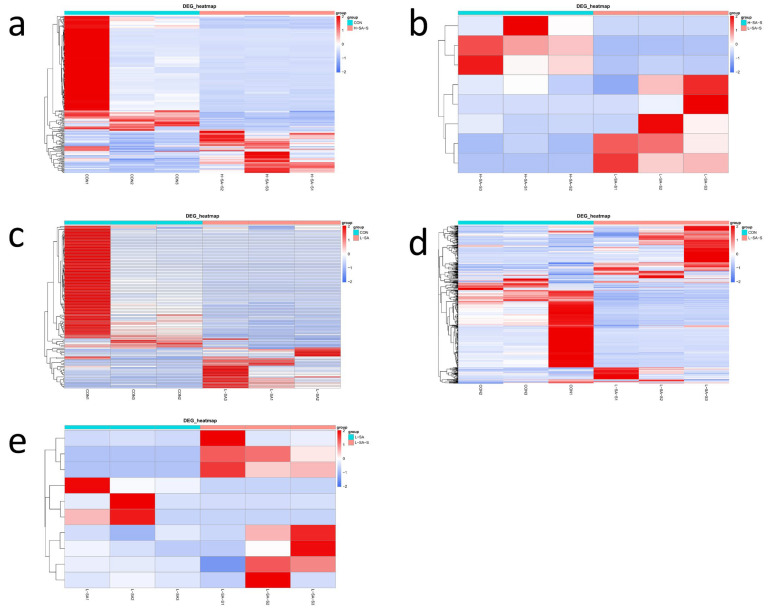
Clustering heatmap analysis of DEGs in different groups ((**a**): H-SA-S vs. CON; (**b**): H-SA-S vs. L-SA-S; (**c**): L-SA vs. CON; (**d**): L-SA-S vs. CON; (**e**): L-SA-S vs. L-SA). Color gradient definition: blue = low relative gene expression; red = high relative gene expression. Similar color blocks indicate high transcriptional correlation between samples.

The heatmap analysis based on hierarchical clustering clearly revealed the differences in transcription characteristics between the experimental groups (Figure 4) and accurately reflected the differential response to the saline–alkaline gradient (high/low) and hydraulic conditions (still water/flowing water system). Notably, the subtle variation observed among biological replicates in the clustering model is likely due to the inherent biological differences among the sampled individuals.

### 3.3. Functional Enrichment Analysis of Differentially Expressed Genes

**Figure 5:**
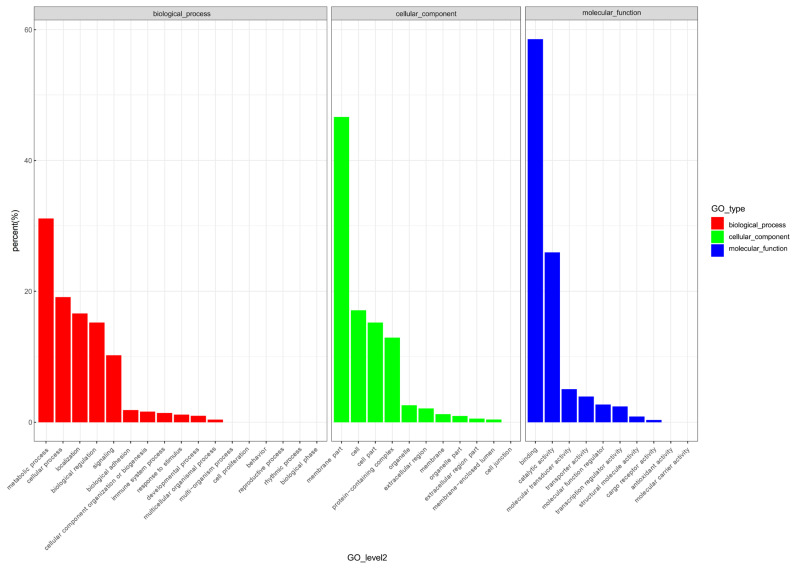
GO functional enrichment classification of all identified DEGs. Top 30 significantly enriched terms are displayed for Biological Process, Cellular Component, and Molecular Function subcategories (*p* < 0.05). X-axis represents the percentage of DEGs enriched in each GO term.

To further explore the biological functions of the DEGs, we performed GO annotation analysis on the DEGs in the L-SA-S, L-SA, H-SA-S, and control groups. The DEGs were annotated according to the three functional branches: biological process, cellular component, and molecular function. The most highly differentially expressed genes were involved in carbohydrate metabolic processes, cellular processes, and localization. For the cellular component annotations, the most highly differentially expressed genes were involved in the membrane, cell, and cell parts. The molecular functions, binding affinity, catalytic activity, and molecular transduction activity were the most enriched among the DEGs (Figure 5).

**Figure 6:**
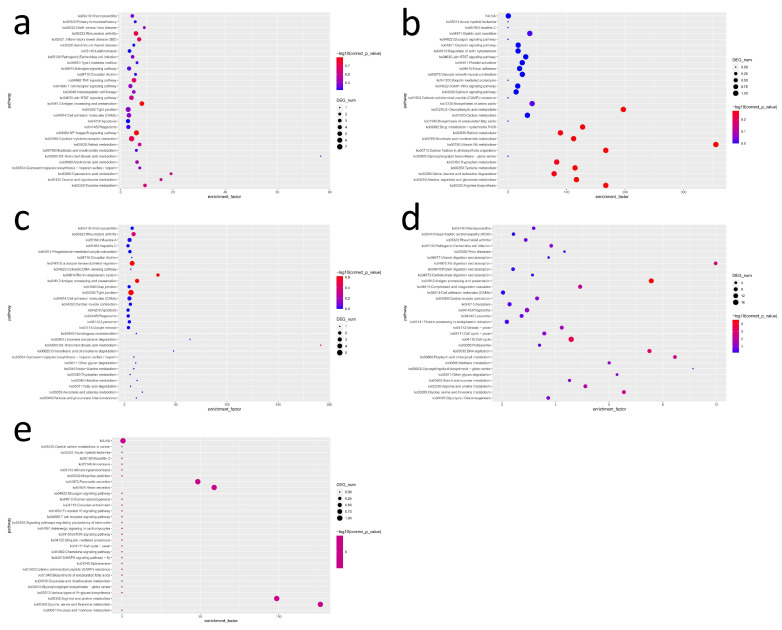
KEGG enrichment scatter plots of hepatic DEGs under combined salinity–alkali and water flow stress. (**a**) Scatter plot of H-SA-S vs. CON; (**b**) Scatter plot of H-SA-S vs. L-SA-S; (**c**) Scatter plot of L-SA vs CON; (**d**) Scatter plot of L-SA-S vs. CON; (**e**) Scatter plot of L-SA-S vs. CON. X-axis = enrichment ratio (higher values mean stronger enrichment); bubble size = number of enriched DEGs within each pathway; bubble color = enrichment significance (blue represents the highest statistical significance, orange the lowest). Each bubble corresponds to one annotated KEGG metabolic/signaling pathway.

The 544 DEGs identified in the comparison of H-SA-S vs. control were further annotated in known KEGG pathways. Among the top 30 pathways, rheumatoid arthritis (ko05323), antigen processing and display (ko04612), NF-κB signaling (ko04064), TNF signaling (ko04668), inflammatory bowel disease (IBD) (ko05321), and cytokine–cytokine receptor interaction (ko04060) were the most significantly enriched pathways (*p* < 0.05). In the comparison of H-SA-S vs. L-SA-S, 41 DEGs were annotated in known KEGG pathways. Among the top 30 pathways, 2-oxocarboxylic acid metabolism (ko01210), drug metabolism-cytochrome P450 (ko00982), retinol metabolism (ko00830), nicotinic acid ester and nicotinamide metabolism (ko00760), vitamin B6 metabolism (ko00750), carbon fixation in photosynthetic organisms (ko00710), tryptophan metabolism (ko00380), tyrosine metabolism (ko00350), valine, leucine and isoleucine degradation (ko00280), alanine, aspartic acid and glutamic acid degradation (ko00250), and arginine biosynthesis (ko00220) had the most significant enrichment (*p* < 0.05). In the comparison of L-SA vs. control, 47 DEGs were annotated in known KEGG pathways. Among the top 30 pathways, leukocyte trans endothelial migration (ko04670), the renin–angiotensin system (ko04614), antigen processing and display (ko04612), rheumatoid arthritis (ko05323), and tight junction (ko04530) were most significantly enriched (*p* < 0.05). In the comparison of L-SA-S vs. control, 1096 DEGs were annotated in known KEGG pathways, of which the complement and coagulation cascade (ko04610), fat digestion and absorption (ko04975), DNA replication (ko03030), and the cell cycle (ko04110) were the most significantly enriched (*p* < 0.05). In the comparison of L-SA-S vs. L-SA, 218 DEGs were annotated in known KEGG pathways, among which pancreatic secretion (ko04972), renin secretion (ko04924), arginine and proline metabolism (ko00330), and glycine, serine, and threonine biosynthesis (ko00260) were among the top 30 pathways (Figure 6).

### 3.4. Validation Using Quantitative Real-Time PCR (qRT-PCR)

To evaluate the reliability of transcriptome data, real-time fluorescence quantitative PCR analysis was performed to quantify the mRNA levels of six genes. Consistent with the transcriptome data, the mRNA levels of *LOC107575423* were upregulated, while the expression of the remaining genes was downregulated in the liver of *Aspiorhynchus laticeps* (Table 4).

## 4. Discussion

### 4.1. Effects of Hydrodynamics on Physiological Regulation

Water flow is a key factor that shapes fish physiological homeostasis and transcriptional adaptation [17]. In the pairwise comparison between the L-SA and L-SA-S groups, the renin secretion pathway (ko04924) was significantly enriched (*p* < 0.001, FDR = 0.012), demonstrating that altered water flow velocity may reshape swimming metabolism and disrupt internal osmotic and energy homeostasis in *A. laticeps* [18]. García Vega et al. reported that flow variation prompts fish to switch from steady sustained swimming to sprinting, which is closely linked to muscle energy storage [19]. The pancreatic secretion signaling pathway (ko04972) exhibited the most prominent enrichment under hydrodynamic variation, indicating that sustained water flow stress may stimulate massive secretion of digestive hydrolases, including trypsin, to maintain feeding efficiency [20]. This regulatory pattern aligns with the findings of Qin, who reported that moderate water flow promotes pancreatic exocrine secretion and stabilizes nutritional metabolism in *A. laticeps* [21]. Additionally, the arginine and proline metabolism pathway (ko00330) was markedly activated in flow-stressed groups. Proline serves as a direct energy substrate under exercise pressure, while arginine can contribute to the tricarboxylic acid cycle to replenish energy consumption [22]. Persistent activation of this amino acid metabolic cascade disrupts baseline energy balance and interferes with core physiological processes such as immunity and osmotic regulation [23,24]. Collectively, hydrodynamic stress remodels energy metabolism via key genes including *NEHOM01_1600* and *gptl*. Although water flow disturbance imposes extra metabolic burdens on *A. laticeps*, it appears to act as a secondary pressure factor rather than the primary driver of population decline for this endangered species.

### 4.2. Metabolism–Immunity Interaction Mechanisms Under Saline–Alkaline Stress

Salinity–alkali stress is a major environmental threat limiting the survival and population recovery of plateau schizothoracin fishes [25,26]. The liver acts as a central organ that coordinates systemic metabolism and innate immunity in teleosts, undertaking carbohydrate, lipid, and protein anabolism and catabolism [27]; thus, hepatic transcriptional profiles can provide important insight into organismal stress resistance capacity [28]. This study systematically characterized hepatic transcriptomic responses of *A. laticeps* under single salinity–alkali stress and combined salinity–alkali plus water flow stress, revealing distinct molecular regulatory hierarchies between the two stress modes. In the comparison of H-SA-S vs. L-SA-S, most enriched KEGG terms centered on basal metabolism, including lipid, amino acid, vitamin, and energy turnover pathways, proving that salinity–alkali exposure fundamentally disturbs metabolic homeostasis. This finding is consistent with the research results of Dietmar and Schreck et al., who reported that saline–alkali stress can cause metabolic disorders and disease in fish [29,30]. Under combined high salinity–alkali and high-water flow (H-SA-S vs. CON), 544 DEGs were identified, with predominant enrichment in immune signal transduction pathways. The TNF/NF-κB signaling pathway (ko04064) was the most highly enriched immune cascade: extracellular TNF ligands trigger intracellular signal cascades that ultimately activate the NF-κB transcription factor family, which governs inflammatory mediator release and immune cell activation [31]. The NF-κB transcription factor family plays key roles in regulating the immune response and inflammation [32]. Prolonged salinity–alkali stress causes excessive activation of the NF-κB pathway, leading to an uncontrolled inflammatory response and non-specific immune attack against healthy hepatic tissues [33]. Chronic immune overactivation induces severe liver tissue damage, which partially contributes to the severe population decline of wild *A. laticeps*. Key stress-responsive genes involved in this immune-metabolic crosstalk include *MST1*, *LOC107702867,* and *LOC113110979*. High salinity–alkali stress markedly upregulated these gene transcripts, thereby enhancing the stress resistance and immune defense capacity of *A. laticeps*. This response represents a core molecular adaptive strategy of *A. laticeps* under combined aquatic stress [34]. In the L-SA-S vs. CON contrast, the complement and coagulation cascade pathway (ko04610) was significantly upregulated. The complement system constitutes the first line of teleost innate defense; transient activation enhances stress resistance capacity, whereas sustained deposition of complement fragments on hepatocyte membranes generates membrane attack complexes that destroy cell integrity [35]. At the transcriptional level, upregulated pro-apoptotic genes trigger hepatic inflammation and organ dysfunction, ultimately elevating mortality risk for stressed *A. laticeps*.

### 4.3. Ecological Cascade Effects and Population Decline

Salinity reshapes plankton community structure in aquatic ecosystems [36]. *A. laticeps* primarily consumes diatoms and green algae, and the growth of this important food source for fish and shrimp (phytoplankton) is inhibited. Stankovic et al. reported that high salinity–alkali conditions severely suppress phytoplankton growth and nutrient absorption efficiency (nitrogen, phosphorus) [37]. Reduced primary productivity diminishes the biomass and diversity of intermediate prey organisms, simultaneously lowering the intrinsic salinity–alkali tolerance of *A. laticeps* and creating dual food shortage and ion stress pressure. This reduces the abundance and diversity of small fish and shrimp prey species, which in turn lowers the salinity–alkalinity tolerance of *A. laticeps* and diminishes its food supply [38]. When salinity–alkali stress co-occurs with unfavorable hydrodynamic conditions, the two stressors exert synergistic inhibitory effects on *A. laticeps* populations: salinity–alkali impairs immune and metabolic homeostasis, while abnormal water flow exacerbates energy consumption, jointly slowing individual growth, reducing physical fitness, and accelerating population decline in wild reserves [39,40]. This multi-stress trophic cascade represents a critical compound factor driving the endangered status of the endemic Tarim River schizothoracin species.

## 5. Conclusions

This study systematically compared hepatic transcriptome profiles of endangered *Aspiorhynchus laticeps* under single salinity–alkali stress, single water flow stress, and combined dual stress and identified a total of 1847 DEGs, which were primarily enriched in immune signal transduction, substance metabolism, and energy homeostasis pathways. Distinct molecular adaptive patterns exist under single and combined stress: single salinity–alkali stress mainly triggers a mild innate immune response with limited DEG variation, whereas combined high salinity–alkali plus high-water flow stress strongly activates the TNF/NF-κB inflammatory immune pathway via upregulating *MST1*, *LOC107702867* and *LOC113110979*, thereby enhancing stress resistance. In contrast, water flow remodels energy metabolism through *NEHOM01_1600* and *gptl* to meet increased swimming-related energy demands. The apolipoprotein gene *LOC107575423* serves as a core dual-stress marker that coordinates lipid metabolism, antioxidants, and immune responses.

Transcriptional evidence from this study clarifies the divergent molecular adaptation strategies of plateau schizothoracin fishes in response to salinization and hydrological alteration in the Tarim River basin. Although this study was limited to short-term acute stress tests on *A. laticeps* under laboratory conditions, our results provide practical molecular-level guidance for wild population habitat restoration, screening of stress-resistant germplasm for artificial breeding, and precise conservation management of endemic, endangered *A. laticeps*.

## Figures and Tables

**Table 1:** Primer used for RT-qPCR.

Gene	Sequence (5′-3′)
NEHOM01_1600	F:GAGTACCCAACAGACCAGCT R:ACGGTGACTTCAAGCAGAGA
LOC107702867	F:CTGTCAGATCAATGTGGCCG R:GCTTTAGTTGCCTCTGAGCC
LOC113110979	F:TGCTGGACAAGGATGGACAT R:CTCATCCTCATCATCGCCCT
*gptl*	F:GGGTCCCGAGTACTTCAACA R:GAACGTGTGCTCTTCTGGTC
LOC107575423	F:CACTCACTGTTTTGCTGGCA R:GTTTGGAAGCCGCTCTGAAA
MST1	F:CTGGGTGCTGAAGAAGAGGA R:AGGGCTGAAAATGGCAAAGG
EF-1α	F:CTTCTTGATGCCCTGGATGC R:AGACTCGTGGTGCATCTCAA

*NEHOM01_1600*, myosin heavy chain 9/10/11/14; *LOC107702867*, disintegrin and metalloproteinase domain-containing protein 9; *LOC113110979*, novel protein kinase C delta type; *gptl*, glutamic–pyruvic transaminase-like; *LOC107575423*, apolipoprotein A-I-1-like; *MST1*, macrophage stimulating 1; *EF-1α*, eukaryotic translation elongation factor 1. The relative expression of target genes versus the eef1a1 was calculated according to the formula: R = 2^−ΔΔCt^.

**Table 2:** Transcriptome sequencing data statistics.

Sample-Name	Reads-Number	Reads-Length	Total-Base	Q20 (%)	Q30 (%)	GC (%)
CON1	38,823,066	150	5,823,459,900	96	89	43
CON2	39,523,252	150	5,928,487,800	96	89	45
CON3	54,618,086	150	8,192,712,900	95	86	45
H-SA-S1	55,286,312	150	8,292,946,800	95	87	46
H-SA-S2	58,637,532	150	8,795,629,800	95	87	46
H-SA-S3	37,406,962	150	5,611,044,300	96	89	46
L-SA1	36,077,826	150	5,411,673,900	96	90	45
L-SA2	41,263,062	150	6,189,459,300	96	89	44
L-SA3	56,036,160	150	8,405,424,000	95	86	46
L-SA-S1	38,426,334	150	5,763,950,100	96	90	48
L-SA-S2	38,304,982	150	5,745,747,300	96	89	47
L-SA-S3	36,847,090	150	5,527,063,500	96	89	46

CON, freshwater static water treatment group; H-SA-S, high-salinity–alkali/high water flow treatment group; L-SA, low-salinity–alkali treatment group; L-SA-S, low-salinity–alkali/high water flow treatment group. Q20: proportion of bases with sequencing accuracy ≥ 99%; Q30: proportion of bases with sequencing accuracy ≥ 99.9%; GC: GC base ratio of clean reads.

**Table 3:** Summary of annotations of differentially expressed genes in different databases.

Date Base	Annotated_Number	Annotated_Ratio
COG	6422	13%
GO	18,241	38%
KEGG	17,337	36%
KOG	21,283	44%
NR	37,202	78%
PFAM	28,806	60%
Swiss prot	28,920	60%
TrEMBL	35,772	75%
Total	37,547	78%

COG: Cluster of Orthologous Groups of proteins; GO: Gene Ontology; KEGG: Kyoto Encyclopedia of Genes and Genomes; KOG: EuKaryotic Orthologous Groups; NR: NCBI Non-redundant Protein Database; PFAM: Protein Family Database; Swiss prot: Manually Annotated Protein Sequence Database; TrEMBL: Automated Annotated Protein Database.

**Table 4:** Relative expression levels of candidate DEGs validated by qRT-PCR.

Expression Level	L-SA vs. CON	L-SA-S vs. CON	H-SA-S vs. CON	H-SA-S vs. L-SA-S
NEHOM01_1600	0.694 ± 0.094 ^b^	23.591 ± 1.842 ^a^	0.784 ± 0.014 ^b^	0.033 ± 0.002 ^b^
LOC107702867	0.330 ± 0.056 ^b^	1.994 ± 0.246 ^a^	0.241 ± 0.027 ^b^	0.122 ± 0.021 ^b^
LOC113110979	0.387 ± 0.018 ^b^	3.614 ± 0.576 ^a^	0.656 ± 0.06 ^b^	0.186 ± 0.042 ^b^
*gptl*	0.795 ± 0.043 ^b^	3.324 ± 0.102 ^a^	0.284 ± 0.028 ^c^	0.085 ± 0.007 ^d^
LOC107575423	13.463 ± 1.263 ^a^	1378.105 ± 83.673 ^c^	227.059 ± 46.109 ^b^	0.164 ± 0.024 ^a^
MST1	2.530 ± 0.191 ^b^	14.146 ± 0.436 ^c^	0.280 ± 0.038 ^a^	0.020 ± 0.002 ^a^

Different lowercase letters indicate significant differences at *p* < 0.05 according to Tukey’s HSD test.

## Data Availability

Because the project has not been finalized, a link to the data has not been made public.
